# The N-fixing legume *Periandra mediterranea* constrains the invasion of an exotic grass (*Melinis minutiflora* P. Beauv) by altering soil N cycling

**DOI:** 10.1038/s41598-019-47380-5

**Published:** 2019-07-30

**Authors:** Carina B. Nogueira, Esther Menéndez, Martha Helena Ramírez-Bahena, Encarna Velázquez, Álvaro Peix, Pedro F. Mateos, Maria Rita Scotti

**Affiliations:** 1 Department of Botany, Institute of Biological Science/Federal University of Minas Gerais, Belo Horizonte, Brazil; 2 Departamento de Microbiología y Genética and Instituto Hispanoluso de Investigaciones Agrarias (CIALE), Universidad de Salamanca, Salamanca, Spain; 3 Instituto de Recursos Naturales y Agrobiología (IRNASA-CSIC), Cordel de Merinas 40-52, 37008 Salamanca, Spain; 4 Unidad Asociada Universidad de Salamanca- CSIC ‘Interacción Planta-Microorganismo’, Salamanca, Spain; 5 Present Address: ICAAM (Institute of Mediterranean Agriculture and Environmental Sciences), University of Évora-Núcleo da Mitra, Évora, Portugal

**Keywords:** Biogeochemistry, Microbiology, Environmental sciences, Plant sciences

## Abstract

*Melinis minutiflora* is an invasive species that threatens the biodiversity of the endemic vegetation of the *campo rupestre* biome in Brazil, displacing the native vegetation and favouring fire spread. As *M. minutiflora* invasion has been associated with a high nitrogen (N) demand, we assessed changes in N cycle under four treatments: two treatments with contrasting invasion levels (above and below 50%) and two un-invaded control treatments with native vegetation, in the presence or absence of the leguminous species *Periandra mediterranea*. This latter species was considered to be the main N source in this site due to its ability to fix N_2_ in association with *Bradyrhizobia* species. Soil proteolytic activity was high in treatments with *P. mediterranea* and in those severely invaded, but not in the first steps of invasion. While ammonium was the N-chemical species dominant in plots with native species, including *P.mediterranea*, soil nitrate prevailed only in fully invaded plots due to the stimulation of the nitrifying bacterial (AOB) and archaeal (AOA) populations carrying the *amoA* gene. However, in the presence of *P. mediterranea*, either in the beginning of the invasion or in uninvaded plots, we observed an inhibition of the nitrifying microbial populations and nitrate formation, suggesting that this is a biotic resistance strategy elicited by *P. mediterranea* to compete with *M. minutiflora*. Therefore, the inhibition of proteolytic activity and the nitrification process were the strategies elicited by *P.mediterranea* to constrain *M.munitiflora* invasion.

## Introduction

**Figure 1:**
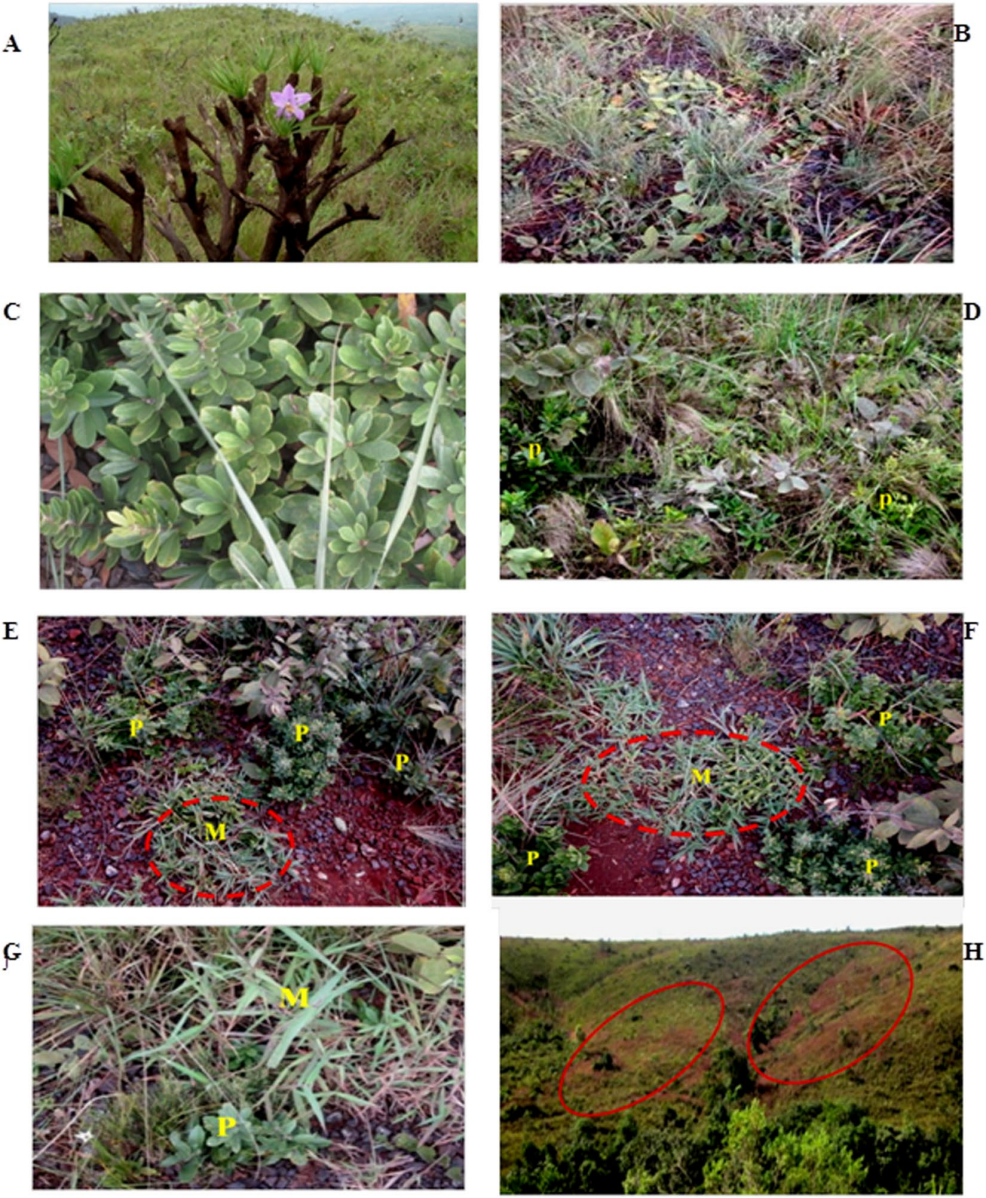
(**A**) Campo Rupestre vegetation; (**B**) (T1) native vegetation without *Periandra mediterranea*; (**C**) *P. mediterranea*; (**D**) (T2) native vegetation with: *P. mediterranea* (P); (**E**,**F**) (T3) beginning of *Melinis minutiflora (M)* invasion; barrier or halo formed by *P. mediterranea* (P); (**G**) *Melinis minutiflora (M)*, >*50%* of invasion; (**H**) *Melinis minutiflora* spots in the field circumscribed by native vegetation. All the photographs were taken by M.R.Scotti.

The Serra do Rola Moça State Park (PESRM), located in the state Minas Gerais (Brazil), covers an area of 3,924 ha and hosts one of the most important preserved areas of the *campo rupestre* biome. This site is formed by ironstone outcrops (canga), with shallow soils resulting from rock dissolution via weathering and/or lichenic acid production^1^. These ironstone outcrops support a notable vegetation at elevations above 1,000 m, with a predominance of grassy, herbaceous and shrub species with a high degree of endemism^2^ (Fig. 1A)

However, this *campo rupestre* vegetation has been massively (60%) invaded by the exotic grass *Melinis minutiflora*, severely impacting the biodiversity and survival of this biome^3^. In addition, the *M. minutiflora* invasion has favoured the occurrence of periodic fires in the park^3^, creating a positive feedback for plant invasion^4^ and leading to land degradation. Such fires have favoured the recycling of soil nitrogen which became available via burned ash^4^ thereby further stimulating the invader expansion. Due to its potential for fire spread^5^ the presence of *M. minutiflora* has became a threat and concern to local populations that live around the park.

It is well known that plant roots establish a molecular communication with neighboring plants and soil microorganisms via root exudation of a wide range of organic compounds, which mediate root-root and root-microbial communication^6^, known as rhizospheric effect^7^. This metabolomic plataform production is plant-specific^8,9^ and mediates the molecular dialogue among co-existing plants by stimulating or inhibiting (allelopathic pathways) the neighboring biological activity^10^. Invasive species can disrupt local feedback mechanisms established in the climax soil system^11^, altering litter decomposition patterns^10,11^ and rhizosphreric microbiome structure and activity^10^. In consequence occurs alterations in nutrient cycling and availability, especially nitrogen^12^. Feedback between plant invasion and nitrogen cycling is often mediated by an increase in N release from the litter^13^ and alterations in specific soil microbial communities involved in N cycling^3,14–17^. Inorganic N forms, such as ammonium (NH_4_^+^) and nitrate (NO_3_^−^), become available to plants after the mineralization of organic compounds containing N, especially those from leguminous plants, through the action of microbial extracellular enzymes. The first step of this process involves the conversion of organic protein N to amino-acids by the action of soil proteases^18^^.^which is followed by the reduction of amino-acids to NH_4_^+^ by extracellular enzymes. The oxidation of NH_4_^+^ to nitrite (NO_2_^−^) and nitrate (NO_3_^−^) is carried out by aerobic chemo-lithotrophic microorganisms as ammonia-oxidising bacteria (AOB) and ammonia-oxidising archaea (AOA), which possess ammonia monooxygenase genes^19^. According to Leininger *et al*.^20^, the AOA population is the most abundant ammonia-oxidising group in soil ecosystems, and this oxidative pathway is performed in different soil types^21^. Besides, nitrification has been positively correlated with AOA abundance^22^ that also has been associated with plant invasion^17^. One of the most reported strategies elicited by invasive species is the increase in soil nitrate, which has been related with a positive feedback not only for the AOA^17^, but also the AOB nitrifying community^3,15,16^ and or by a negative feedback via the inhibition of the denitrifying microbial community^14^. *Melinis minutiflora* displayed a positive feedback with the nitrogen cycle by increasing nitrate and the AOB-nitrifying community in an invaded *campo rupestre* site^3^. These authors found that the main nitrogen source for the site was represented by biological nitrogen fixation performed by the prevalent leguminous species *Mimosa pogocephala*, which was associated with *Burkholderia nodosa*, assuring soil N fertility.

Therefore, we hypotized that (1): the N-fixing leguminous species are the main N source in the *campo rupestre*, making the ammonium-N, the predominant chemical species available to other plants, and that (2) *M.minutiflora* disrupts the soil N cycling by increasing soil nitrate via a positive feedback over nitrifying microorganisms as a general mechanism of invasion. Thus, our objective was to assess the alterations in the nitrogen cycle promoted by *M. minutiflora* over an iron-rich rocky outcrop (*campo rupestre*) under contrasting invasion degree and in the presence/absence of the e native leguminous species *Periandra mediterranea*. The overall aim of this study was to understand the general mechanism of *M. minutiflora* invasion in order to develop adequate management strategies for the control of this species in the *campo rupestre*.

## Results

### Plant occupation

**Figure 2:**
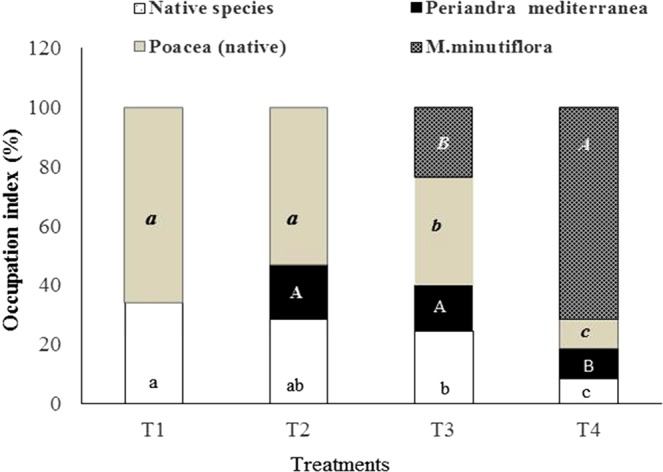
Occupation index of the vegetation in plots as follows: (T1) native species without *Periandra mediterranea* and no invasion; (T2) native species plus *P. mediterranea* and no invasion; (T3) native species plus *P. mediterranea* and ≤50% invasion; (T4) native species plus *P. mediterranea* and >50% invasion. Means with different letters are significantly different according to Kruskal-Wallis and Nemenyi tests at 5% confidence level (p ≤ 0.05).

Figure 2 shows the plant occupation index in plots or treatments. The plots occupied by native species (T1, Fig. 1B) were mainly composed of plants of the Poaceae family (65%), (Fig. 2). In the presence of native species plus the leguminous species *P. mediterranea* (T2, Fig. 1C,D), the proportion of native species remained more or less constant (Fig. 2). At the beginning of the *M.minutiflora* invasion (T3 Fig. 1E,F), all native species were weakly reduced, independent of the plant family (Fig. 2). In T3 plots, we observed a space adjacent to *P. mediterranea*, which keeps this species distant from the invader. We termed this space “barrier” or “halo” (Fig. 1E,F and H), and it disappeared in T4, when the invasion exceeded 50% (Fig. 1G). In T4 (Fig. 2), we observed a decline of native species, especially those from the Poaceae family.

### Leaf C and N concentrations

**Figure 3:**
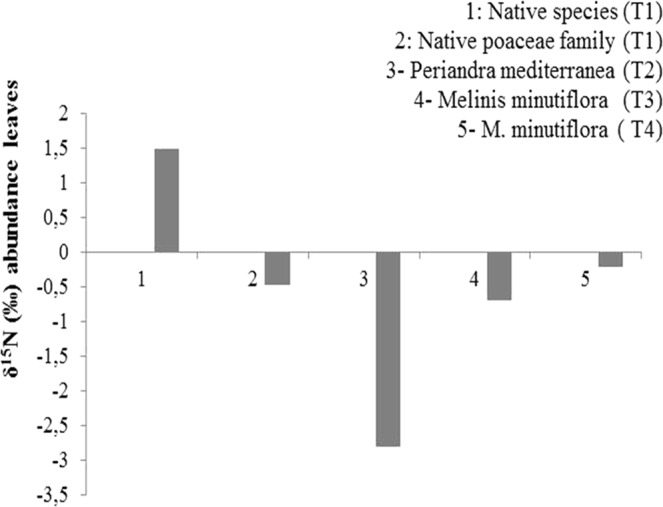
Natural abundance of δ^15^N in leaves of plant species in the treatments as follows: (T1) native species without *Periandra mediterranea* and no invasion with *Melinis minutiflora*; (T2) native species plus *P. mediterranea* and no invasion; (T3) native species plus *P. mediterranea* and ≤50% invasion; (T4) native species plus *P. mediterranea* and >50% invasion.

The leaf C and N concentrations (Table 1) of *P. mediterranea* (T2) were significantly higher than those of the other native (T1) and invasive (T3 and T4) species. Consequently, the C:N ratio in *P. mediterranea* leaves (T2) was the lowest, followed by that of the native species in T1, contrasting with the highest values found in *M. minutiflora* leaves (T3 and T4). The leaf natural ^15^N abundance (‰) of *P. mediterranea* showed a greater isotopic depletion than that of leaves of native and invasive species, suggesting a different nitrogen source among plants (Fig. 3).

**Table 1:** Comparative analysis among all variables (leaf carbon, leaf nitrogen and C:N ratio) and multiple comparisons (pairwise comparisons) ofthe following treatments: (T1) leaves of native species from plots without *Periandra mediterranea* and no invasion; (T2) leaves of *P. mediterranea* in plots with native species and no invasion; (T3) leaves of *Melinis minutiflora* in plots *with* ≤50% invasion, native species and *P. mediterranea*; (T4) leaves of *Melinis minutiflora* in plots with >50% invasion,native species and *P. mediterranea*. Means in bold are significantly different according to the Kruskal-Wallis test at 5% confidence level (p ≤ 0.05) and multiple comparisons by the Nemenyi test.

Treatments		Mean	P-value	P- value -Multiple comparisons			
Leaf carbon (%)	T1	46.68	**0.007**		T1	T2	T3
	T2	48.11		T2	0.535	—	—
	T3	45.02		T3	0.359	**0.015**	—
	T4	44.84		T4	0.411	**0.020**	0.999
Leaf nitrogen (%)	T1	1.37	**0.000**		T1	T2	T3
	T2	1.97		T2	0.138	—	—
	T3	0.89		T3	0.070	**0.000**	—
	T4	0.96		T4	0.335	**0.000**	0.872
Leaf C: N	T1	36.06	**0.000**		T1	T2	T3
	T2	24.71		T2	0.159	—	—
	T3	51.68		T3	0.052	**0.000**	—
	T4	47.35		T4	0.359	**0.001**	0.795

### Identification of rhizobial endosymbionts of *P. mediterranea*

**Figure 4:**
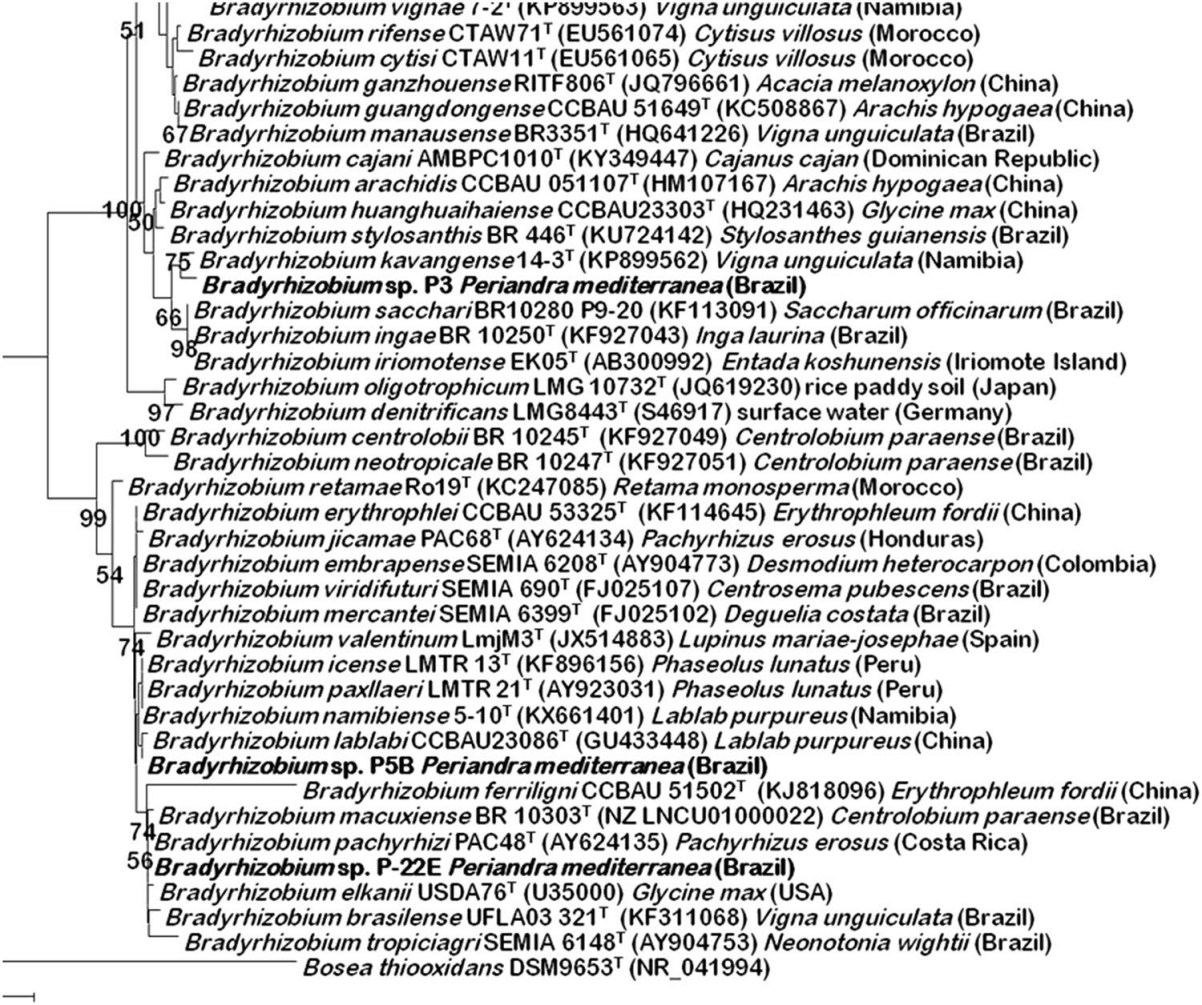
Neighbour-joining phylogenetic tree based on 16S rRNAgene sequences showing the position of the strains isolated from *P. mediterranea* within genus *Bradyrhizobium*. Bootstrap values calculated for 1,000 replications are indicated. Bar, 1 nt substitution per 100 nt.

The slow-growing isolated strains from *P. mediterranea* nodules formed mucoid colonies typical of rhizobia. The 16S rRNA gene sequences of the isolated strains were compared with those kept in EzBiocloud, a database containing the sequences of the 16S rRNA genes of the type strains of all bacterial species, and the results showed that they belonged to the genus *Bradyrhizobium*. Phylogenetic analysis of their 16S rRNA gene sequences showed that they clustered into three different phylogenetic groups within this genus, which also contained other species isolated in Brazil (Fig. 4). This is therefore the first report of the identity and diversity of *Bradyrhizobium* strains inhabiting nodules of *P. mediterranea*.

### Soil N chemical species

**Figure 5:**
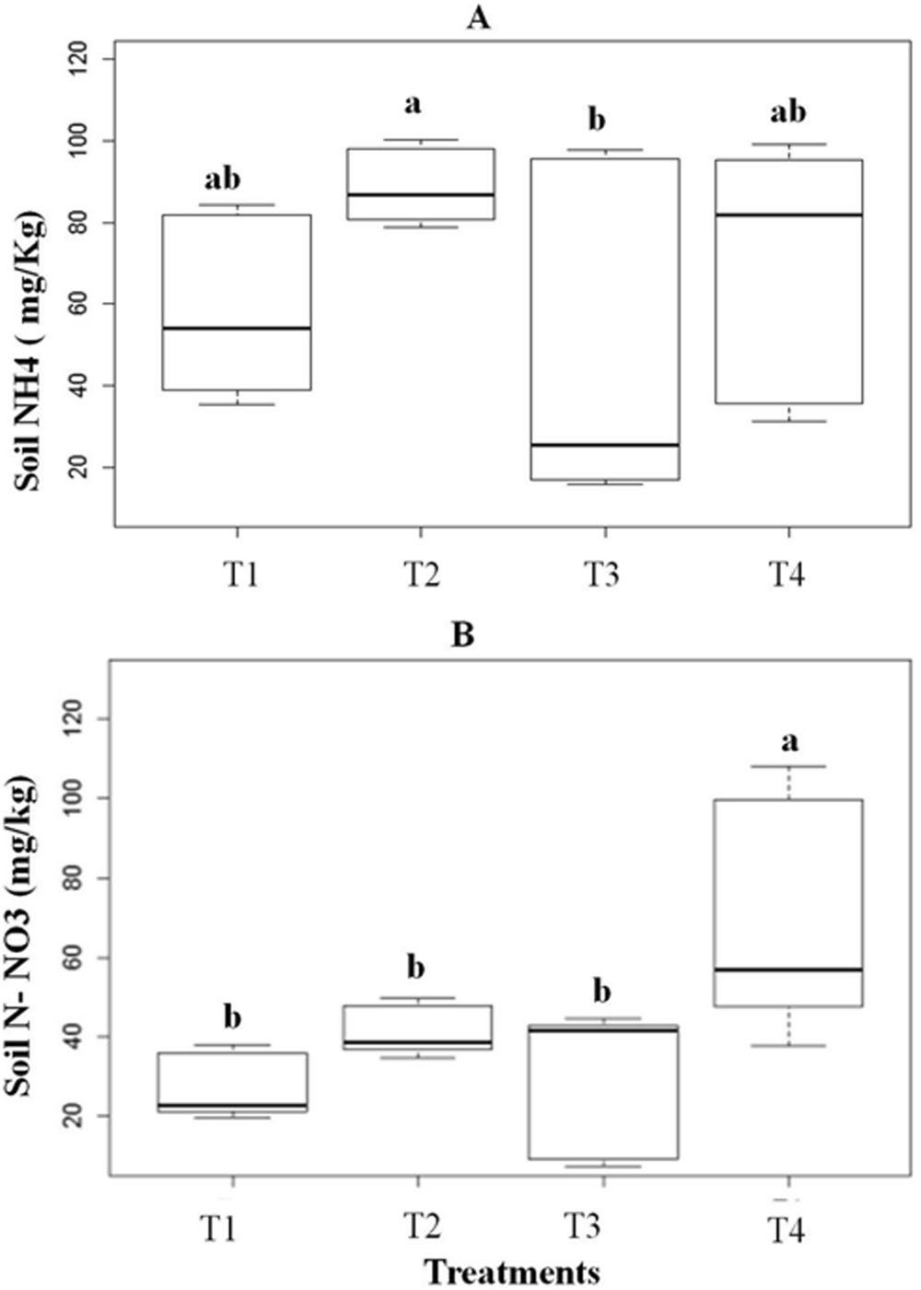
Soil contents of ammonium (**A**) and nitrate (**B**) under different treatments: (T1) native species without *Periandra mediterranea* and no invasion; (T2) native species plus *P. mediterranea* and no invasion; (T3) native species plus *P. mediterranea* and ≤50% invasion by *Melinis minutiflora*; (T4) native species plus *P. mediterranea* and >50% invasion. Means values ± SE (standard error) with different letters are significantly different according to Kruskal-Wallis and Nemenyi tests at 5% confidence level (p ≤ 0.05). NS: No significant differences.

Although the total C and N contents did not differ among treatments (Table 2), the soil C:N ratio was significantly lower in T2 plots, where *P. mediterranea* was the dominant plant species. Soil NH_4_^+^did not differ between the treatment with native plants (T1) and T2 (p = 0.13); T3 (p = 1,0) and T4 (p = 1.0). However, the NH_4_^+^ content was reduced in T3 in relation to T2 (p = 0.01) at the beginning of the invasion (Fig. 5A), but there was no difference between T2 and T4 at advanced invasion (p = 0.9). Indeed, at a higher invasion degree (T4), the NH_4_^+^ content was restored to similar levels as those found before the invasion (T1 and T2). In contrast, soil NO_3_^−^ was significantly increased in T4 (Fig. 5B) when invasion by *M. minutiflora* was highest (Fig. 1G), differing from T1 (p < 0.001), T2 (p = 0.02) and T3 (p < 0.001) (Fig. 5B). Nevertheless, such increase in nitrate did not occur at the initial invasion process (T3), and nitrate levels were similar to both T1 (p = 1.0) and T 2 (p = 1.0). Similarly, the presence of *P. mediterranea* in T2 did not facilitate nitrate formation when compared to the plots only containing other native species (T1) (p = 0.54).

**Table 2:** Comparative analysisof all variables (soil carbon, nitrogen and C:N ratio) among treatments and their multiple comparisons (pairwise comparisons). (T1) native species without *Periandra mediterranea* and no invasion; (T2) native species plus *P. mediterranea* and no invasion; (T3) native species plus *P. mediterranea* and ≤50% invasion; (T4) native species plus *P. mediterranea* and >50% invasion. Means in bold are significantly different according to the Kruskal-Wallis test at 5% confidence level (p ≤ 0.05) and multiple comparisons by the Nemenyi test.

Treatments		Mean	P- value	P-value Multiple comparisons			
Soil carbon (%)	T1	2.43	0.636		T1	T2	T3
	T2	2.54		T2	0.621	—	—
	T3	2.85		T3	0.997	0.745	—
	T4	3.33		T4	0.930	0.930	0.977
Soil nitrogen (%)	T1	0.14	0.337		T1	T2	T3
	T2	0.18		T2	0.311	—	—
	T3	0.17		T3	0.979	0.542	—
	T4	0.20		T4	0.789	0.857	0.950
Soil C:N	T1	16.64	**0.001**		T1	T2	T3
	T2	15.22		T2	**0.001**	—	—
	T3	16.52		T3	0.899	**0.019**	—
	T4	16.34		T4	0.903	**0.019**	0.999

### Soil enzyme activity and microbial nitrifying communities

**Figure 6:**
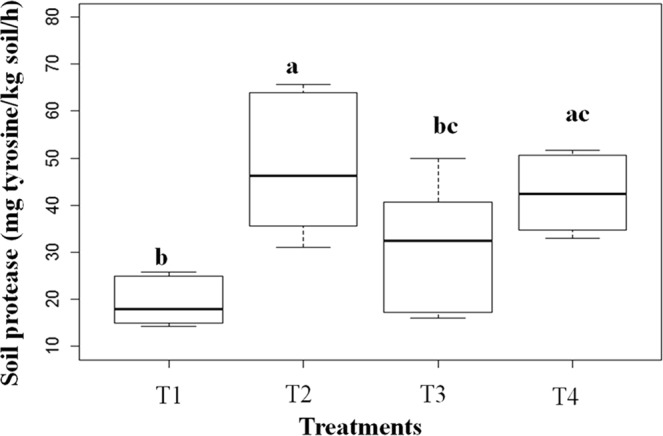
Soil protease activity; for the following treatments: (T1) native species without *Periandra mediterranea* and no invasion; (T2) native species plus *P. mediterranea* and no invasion; (T3) native species plus *P. mediterranea* and ≤50% invasion by *Melinis minutiflora*; (T4) native species plus *P. mediterranea* and >50% invasion. Means values ± SE (standard error) with different letters are significantly different according to Kruskal-Wallis and Nemenyi tests at 5% confidence level (p ≤ 0.05).

Soil enzyme activity related to N metabolism (Fig. 6) showed that protease activity was higher in the presence of the legume *P. mediterranea* (T2) than in the plots without this species T1 (p < 0.001), but was inhibited in the beginning of the invasion process (T3), which differed from T2 (p = 0.012). In the first stage of the invasion (T3), enzyme activity became similar to that in T1 (p = 0.1) plots without *P. mediterranea*. However, in fully invaded plots (T4), we observed a significant increase of soil protease activity in relation to T1 (p < 0.001), but not in relation to T2 (p = 1.0) and T3 (p = 0.19).

**Figure 7:**
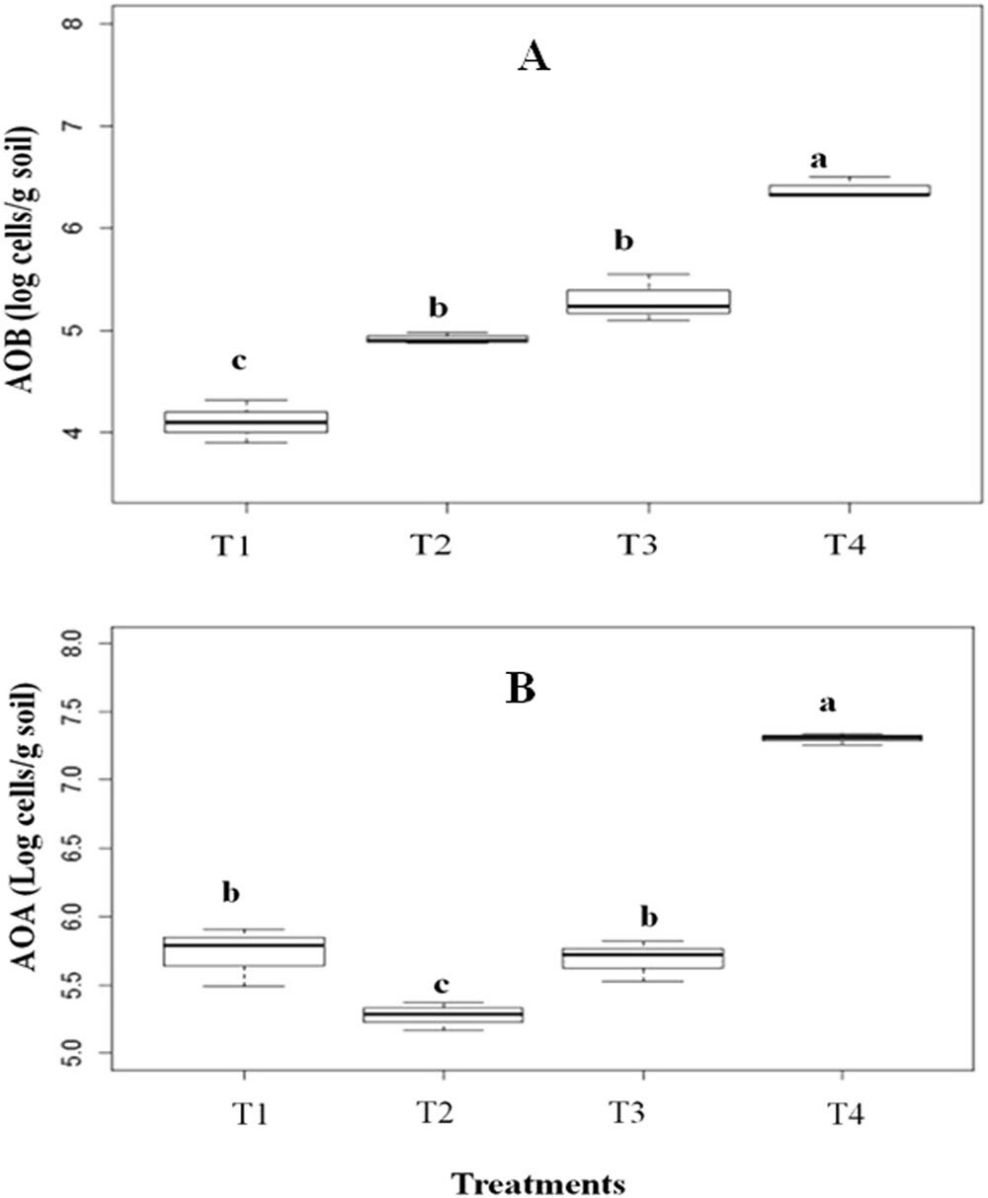
(**A**) ammonia-monooxygenase abundance of AOB and (**B**) ammonia-monooxygenase abundance of AOA, estimated by qPCR for the following treatments: (T1) native species without *Periandra mediterranea* and no invasion; (T2) native species plus *P. mediterranea* and no invasion; (T3) native species plus *P. mediterranea* and ≤50% invasion by *Melinis minutiflora*; (T4) native species plus *P. mediterranea* and >50% invasion. Means values ± SE (standard error) with different letters are significantly different according to Kruskal-Wallis and Nemenyi tests at 5% confidence level (p ≤ 0.05).

The abundance of functional genes of microbial communities that mediate the first step of the nitrification process, oxidation of NH_4_^+^ to NH_2_OH, which is subsequently oxidized to nitrite and nitrate, was estimated by quantifying the *amoA* gene carried by bacteria (AOB) and archaea (AOA) communities (Fig. 7A,B). In soils containing native plants (T1), we detected a low abundance of bacterial (AOB) *amoA* gene communities (Fig. 7A), which was increased in the presence of *P. mediterranea*, either in T2 (p = 0.0018) or T3 (p < 0.001). In contrast, in the initial stages of the invasion process (T3), the AOB communities were not modified by *M. minutiflora* in comparison to T2 (p = 0.14), but they were significantly stimulated with the dominance of the invasive plant in T4 in relation to T1 (p < 0.001), T2 (p < 0.001) and T3 (p = 0.002) (Fig. 7A). Regarding the AOA nitrifying communities (Fig. 7B), there was an inhibition of these microbial communities in the treatment with *P. mediterranea* (T2) in comparison to the native species (T1) (p = 0.001), but they were progressively stimulated by the invasive species (T3), with a significant difference to T2 (p = 0.001), but not to T1 (p = 1.0). The highest abundance of AOA was registered in T4 as compared toT1 (p < 0.001), T2 (p < 0.001) and T3 (p < 0.001).

## Discussion

The occupation data show a displacement of native species, particularly those belonging to the Poaceae family, which were replaced by *M. minutiflora*, also a Poaceae species. Such replacement of native plants by the invader might be due to competition for nutrients such as nitrate, as revealed by our results and confirmed by the literature, which demonstrates that *M. minutiflora* displays a high demand for nitrogen^3,4.5^. In contrast, *P. mediterranea* suffered a less significant displacement than the other plants in T3, possibly due to its independence for soil nitrogen uptake associated with its nitrogen fixing ability.

Our results demonstrate that the main N source in the studied site is represented by biological nitrogen fixation via *P. mediterranea*, which showed the highest total leaf N concentration, the lowest leaf C:N ratio and, most importantly, a depletion in leaf isotopic ^15^N relative to other reference species. Such depletion is attributed to the isotopic atmospheric discrimination promoted by N-fixing bacteria associated with leguminous plants^23^. In this study, *P. mediterranea* was found nodulated by three species of the symbiotic nitrogen-fixing genus *Bradyrhizobium*, some of them clustering with species isolated from nodules of other Brazilian legumes, such as *Bradyrhizobium ingae* (*Inga laurina*), *Bradyrhizobium tropiciagri* (*Neonotonia wightii*), *Bradyrhizobium macuxiense* (*Centrolobium paraense*) and *Bradyrhizobium brasilense* (*Vigna unguiculata*). Besides, a novel bacterial species was found inhabiting the nodules of *P. mediterranea* and belonging to the genus *Paenibacillus* (*P. periandrae*)^24^. This bacterial species might also play a role on nitrogen fixation, since the genus *Paenibacillus* contains several nitrogen-fixing species^25^.

The N fixation occurring in *P.mediterranea* nodules explains the elevated N content (2.0 g/kg) in plots with this species, which is similar to the values (2.0 g/Kg) found in tropical soils in Brazil, such as in the Atlantic^26^ and the Amazon^27^. The C:N ratio is an important tool to assess soil fertility in both agricultural and natural ecosystems^28^. Although total soil N and C did not differ among treatments, the C:N ratio was lowest in T2. Therefore, the presence of *P. mediterranea* seems to ensure N soil fertility in study area, which may be considered the main N source. Since soil N becomes available via mineralisation, we infer that not only native species use it, but that it can be opportunistically shared with invasive species, supporting the “Fluctuating Resource Hypothesis” for plant invasion^29^. Although total soil N was not altered by plant invasion, *P. mediterranea* stimulated the proteolytic activity in the soil, especially in T2 (*P.mediterranea* + native species) in relation to T1 (native species). Similar to T1, the proteolytic activity in T3 (*P. mediterranea* + native species + invasion < 50%) was limited in relation to T2, suggesting the establishment of a competitive relationship between the leguminous and the invasive species to resist the invasion.

Although the molecular dialogue among co-existing plants involving root-root interactions is not fully understood, root exudates may affect all the components of the rhizobiome^8,9^, allowing the defense of native species by establishing a competition against invaders^11^. Thus, we may infer that the competition established by *P.mediterranea* with the invader may likely be processed by root-root interactions via molecular signalling, able to detect the presence of the invader and illicit a defense response.

However, such competition seems to has been overcome by the invasive species by restoring proteolytic activity when it reached the maximum invasion in T4, possibly also via root exudation, stimulating the proteolitic activity. Since soil protease may be derived either from microbial or plant exudation^30^, the increase in this enzyme in both *P. mediterranea* and in fully invaded *M. minutiflora* plots (T2 and T4) may also be explained by the capacity of these species to act directly via protease exudation or indirectly through the stimulation of decomposing microbial populations, thereby increasing N release^13^. In previous studies, soil protease activity was increased under both leguminous species^31^ and invasive grass species^32^. Similar to our results, these latter authors found a significant increase in protease activity in the rhizosphere of the invasive grass *Pennisetum setaceum* in relation to native species. On the other hand, despite the presence of leguminous species, soil protease was inhibited in T3, which can be explained by the root exudation of protease inhibitors such as flavonoids, plant hormones and siderophores by *P. mediterreanea* or microorganisms^30^, constituting a possible allelopathic defence mechanism at the beginning of invasion.

Proteolysis is a slow and limiting process of the N cycling and represents the natural supply of soil low-molecular-weight monomers (e.g., amino-acids), facilitating mineralisation^33^ and making the inorganic N forms (NH_4_^+^and NO_3_^−^) available to plants. Such availability depends on abiotic factors such as soil pH and on biotic factors such as plant species traits. Ammonium is the dominant soil inorganic N species in most mature undisturbed forests and acidic soils^34–36^, while nitrate is more available in agricultural soils with higher pH levels^34,35^. As under most native vegetation, the dominant chemical N form is NH_4_^+^, it is likely to be the target of competition between plants and microorganisms^36^. Furthermore, plant uptake of inorganic N also depends on the expression of different protein transporters, which are modulated by the soil ammonium/nitrate ratios^34^ determining the N chemical preference by plants in their native habitats^35^.

Considering that the *campo rupestre* biome is a preserved site with acidic soils and elevated NH_4_^+^ content in relation to NO_3_^−^, as found in plots with native species (T1, T2 and T3), the former appears to be the preferred N chemical species naturally used by this vegetation, as also observed by Ribeiro *et al*.^3^. Similar to the protease activity, NH_4_^+^ formation was inhibited in plots weakly invaded by *M. minutiflora* (T3), reinforcing the idea of a competitive interaction between native and invasive species for nutritional resources. Such reduction occurred even in the presence of the N-fixing species *P. mediterranea*. High NH_4_^+^ levels were also found in massively invaded plots (T4), suggesting that both *M. minutiflora* and native species may use NH_4_^+^ as N source. Indeed, Incerti *et al*.^13^ observed a greater N release from litter of invasive species as compared to natives, confirming the high dependence of invasive species on N. Thus, the competitive equilibrium for NH_4_^+^ observed in T3 was disrupted when the invader stimulated the oxidation of NH_4_^+^ to NO_3_^−^(T4), which was found elevated only in the fully invaded plots, suggesting that it was the main N form used by the invasive plants; this has also been observed by Ribeiro *et al*.^3^. However, in the presence of the native leguminous species *M. pogocephala*^3^, nitrification was stimulated in the first stage of the invasion process. In contrast, in the present study, we found low levels of NO_3_ at the beginning of the invasion of *P. mediterranea* plots (T3), confirming the competitive state for N established between native and invasive plants. Such constraint in soil N metabolism elicited by *P. mediterranea* in T3 suggests a possible mechanism of biotic resistance to control exotic species invasiveness, as shown in Fig. 1E,F.

Nitrate formation in the soil is mediated by aerobic ammonia-oxidising bacteria (AOB)^37^ or by ammonia-oxidising archaea (AOA) carrying *amo*A genes^38^. In the *campo rupestre*, an increase in both bacterial and archaeal populations (AOB and AOA) was found, as expressed by the *amo*A gene abundance in T4, contrasting with lower levels in plots with native species (T1, T2 and T3). These results showed a high reproducibility in relation to those obtained by Ribeiro *et al*.^3^, who, using the same experimental design in a site separated from the present study site, both spatially (almost 1 Km) and temporally (2 years), also demonstrated that the increase in soil nitrification is the main competitive strategy of *M. minutiflora* to invade the *campo rupestre* biome. Literature findings also confirm a positive feedback between different invasive plants and NO_3_^−^ availability, mediated by both AOA and AOB nitrifying microbial populations^3,15–17^. In addition, Rodríguez-Caballero *et al*.^32^ demonstrated that the nitrifier genus *Ohtaekwangia* was also stimulated in the rhizosphere of the invasive species *Pennisetum setaceum* and Bardon *et al*.^39^ reported a significant increase of nitrate and nitrification enzyme activity in a site invaded by *Pteridium aquilinum*.

However, at the initial steps of invasion (T3), the AOB population was not stimulated and remained similar to that of non-invaded plots (T2), suggesting again a competition between *P. mediterranea* and the invading species involved in the control of the AOB population. This idea is reinforced by the results found by Ribeiro *et al*.^3^ who reported that *M. minutifora* also strongly stimulated the AOB population, although this occurred at the very beginning of the invasion, when the dominant leguminous species was *Mimosa pogocephala*.

In contrast to the AOB population, the AOA population was smaller in the *P. mediterranea* plots (T2) in comparison to those plots without this species (T1) as well as at the beginning of the invasion (T3). Such a high AOA abundance in T1, T3 and T4 in relation to T2 may be attributed to the effect of grassy species, since AOA is particularly favoured in grasslands^40^. Therefore, the increase in AOA abundance at the first steps of this grass invasion process seems to be a key factor favouring *M. minutiflora* to overcome the competition against *P. mediterranea* and successfully invade the site. The inhibition of AOA population in presence of leguminous species has been also reported by Paungfoo-Lonhienne *et al*.^41^ who found that AOA population was suppressed by legumes in a sugarcane cropping soil.

At the beginning of the invasion of *campo rupestre* by *M. minutiflora*, when this plant still co-existed with *P. mediterranea* (T3), soil nitrogen metabolism was limited, as observed for proteolytic activity, ammonium, and nitrate production as well as nitrifying AOB, but not in terms of AOA populations. Such inhibition in N metabolism was observed in the presence of *P. mediterranea*, but not when *M. pogocephala* was the dominant species^3^, suggesting that the former species elicited such an “N metabolic silence” as a strategy to constrain the expansion of the invader. Such strategy may be related to the independence of *P. mediterranea* in relation to soil nitrogen due to its N-fixing ability as well as the control effect over the AOB population and the inhibition of soil protease activity. However, this strategy seems to have been weakened by the stimulation of the AOA community by the grassy invasive plant, enabling it to oxidise ammonium to nitrate, which is the N species found only beneath *M. minutiflora*. Such a positive feedback between invader and soil nitrate allowed this species to successfully invade the *campo rupestre*, as observed in T4.

Therefore, the inhibition of some steps of the N cycle, elicited by *P. mediterranea*, seems to be a strategy of biotic resistance. Traditionally, biotic resistance is considered as the ability of resident species to constrain the invasive success of exotic species^42^ through different mechanisms such as an increase in competition, predation, disease and parasites as well as a decrease in nutrient availability^43^, including N^44^. Considering that the competition for nutritional resources is a major invasive strategy used by exotic species, as proposed by the “Fluctuating Resource Hypothesis”^29^, the unavailability of some soil N chemical species as nitrate, elicited by native plants, might be one of the strategies to retard the invasion, as shown in our study. Thus, at the beginning of the invasion process, *P. mediterranea* limited the availability of all soil N species, but particularly constrained nitrate formation, which possibly constitutes one of the engines of the biotic resistance against *M. minutiflora* invasion in the *campo rupestre* biome. The mechanism driving the biotic resistance has been attributed to the production of plant secondary metabolites as methyl 3-(4 hydrophenyl) propionato or cyclic diterpene able to inhibit specific nitrifies groups^45^ by declining the abundance of both AOA and AOB^46^. Such decrease in ammonia oxidizers population promoted by plants is a conservative strategy to preserve the NH4^+^^47^ a phenomenon so-called as biological nitrification inhibition (BNI) as suggested by Subbarao *et al*.^46^.

In conclusion, this study highlighted the ability of *M. minutiflora* to displace the native species in the *campo rupestre*, particularly those from the Poaceae family, while the leguminous *P. mediterranea* showed some resistance. *P. mediterranea* resistance was attributed not only to its N-soil independence due to biological nitrogen fixation in association with *Bradyrhizobium* species, but also to its ability to compete with *M. minutiflora* at the beginning of the invasion specially via biological nitrification inhibition (BNI). At this stage, there was a constraint of proteolytic activity, ammonium and nitrate formation as well as AOB abundance. However, the invasive species was able to overcome the competition against the native species by stimulating the AOA nitrifying population. Therefore, selection of species that display biotic resistance via nitrification inhibition (BNI) might be a useful strategy to restore *M. minutiflora* invaded lands. Regarding specifically the invaded *campo rupestre* sites by *M.minutiflora*, we highly recommend planting *P.mediterranea* in future rehabilitation efforts.

## Methods

### Study area

The study was developed at the PESRM (3924 ha), which is located in the central-southern region of the state Minas Gerais, Brazil (20°3′20″S,44°1′11″W). The park is inserted in the Espinhaço mountain region, which has a giant hematite-rich iron deposit, largely exploited by iron ore mining companies. The dominant *campo rupestre* vegetation is composed of grassy, herbaceous and shrub species of the families the Poaceae, Asteraceae, Fabaceae, Myrtaceae, Melastomataceae and Orchidaceae^2^. Leguminous species occur frequently, particularly of the genera *Periandra*, *Chamaecrista*, and *Mimosa*^2^. This vegetation grows on shallow soils formed on ironstone outcrops (canga) originated from rock dissolutions, which are also rich in soil organic matter and nitrogen^3^.

### Experimental design

The experimental site was established in an area of 600 m^2^ or 0.06 ha of a grassy field (20°03′22″S,44°01′09″W). The experimental design consisted of three blocks with four treatments (plots), with three replicates/treatment/block (3 × 4 × 3 = 36), resulting in a total of 36 plots of 4 m² each. Each studied plot (4 m^2^) was circumscribed by a 1-m strip with the same treatment, defining a large plot (3 × 3 m) to control the edge effects. Each plot (4 m^2^) represented one treatment. Three soil sub-samples/ treatment/ block were collected, resulting in a total of nine samples/treatment.

In the study site (Fig. 1), four treatments were established: T1- Native vegetation without the legume species (*P. mediterranea*) (Fig. 1B) and no invasion; T2 - Native vegetation plus *P. mediterranea* and no invasion. (Fig. 1C,D); T3 - Native vegetation plus *P. mediterranea* and invasion by *M. minutiflora* ≤50% (Fig. 1E,F); T4 - Native vegetation plus *P. mediterranea* and invasion by *M. minutiflora* >50% (Fig. 1G,H). After the demarcation of the plots, species occupation and occurrence were measured using the method proposed by Toledo and Schultze-Kraft^48^. This method uses a quadrant of 1 m², composed of 100 squares of 0.01 m² each. The quadrant was placed inside the plots and all plant species inside each square were identified.

### Soil chemical analysis and soil protease activity

Each soil sample consisted of three mixed subsamples per treatment/block or nine mixed samples per treatment. Total inorganic nitrogen was determined via semi-micro-Kjeldahl digestion^49^, and N ammonia and nitrate contents were determined^50^. Soil protease activity was determined by the method described by Alef and Nannipieri^51^ and expressed as mg tyrosine g^−1^ h^−1^.

### Plant leaf analysis

Leaf samples were taken in the field, using three replicates from three individuals of each family or species (Poaceae families; native species belonging to Malpiguiaceae, Asteraceae, Melastomataceae, Euphorbiaceae and Cactaceae families; *P. mediterranea* and *M. minutiflora*) per treatment/block and dried at 70 °C for at least 48 h. After reaching constant weight, the samples were ground to a fine powder in a ball mill (Glen Creston Ltd.) and subsequently analysed for total carbon, nitrogen, and δ^15^N, using an isotope ratio mass spectrometer (Finnigan MAT Delta E, Thermo Electron, Bremen, Germany) coupled to an EA 1110 elemental analyser (Thermo Electron, Milan, Italy). The abundance of ^15^N was expressed in “delta” notation (δ), which is the deviation per thousand (‰) of ^15^N abundance of the sample in relation to the international standard (i.e. atmospheric N_2_, which has an atom% of ^15^N = 0.3663^52^:

$${{\rm{\delta }}}^{15}{\rm{N}}\,(\textperthousand )=(\frac{{\rm{sample}}\,{\rm{atom}}{ \% }^{{\rm{15}}}{\rm{N}}\mbox{--}{\rm{0.3663}}}{0.366})\times {\rm{1000}}$$

### Identification of rhizobial endosymbionts of *P. mediterranea*

The isolation of rhizobia was carried out using *P. mediterranea* effective nodules (internal pink colour) on YMA medium according to Vincent^53^. Identification was performed through the analysis of the 16S rRNA genes, which were amplified^54^. Nearly complete sequences (more than 1,400 bp) were obtained by the Macrogen Corporation (Amsterdam, The Netherlands). The sequences obtained were compared to those stored in the EzBiocloud server^55^. For the phylogenetic analysis, the sequences were aligned by using the Clustal W software. Distances were calculated according to Kimura’s two-parameter model^56^ and used to infer phylogenetic trees with the neighbour-joining method and the MEGA5 software^57^. Confidence values for nodes in the trees were generated by bootstrap analysis using 1,000 permutations of the data sets.

### AOA and AOB abundances (qPCR)

The abundances of AOB and AOA communities were determined using real-time PCR (qPCR) to estimate the soil bacteria expressing the ammonia monooxygenase gene (*amo*A). The DNA was extracted from three mixed soil samples/treatment (0.5 g), using the MoBio Power Soil extraction kit (Carlsbad, CA) and following the recommended protocol. The DNA concentration and purity measurements were performed using a Nanodrop spectrophotometer (Nano Drop Technologies, Wilmington, DE, USA), and the PCR reactions were carried out in an Applied Biosystems Quant Studio Real-Time PCR system (Applied Biosystems, Foster City, CA) with iQSYBR-Green Supermix from Bio-Rad (Hercules, CA). Bacterial *amoA* gene quantification was performed using the primers *amoA-*1F (5′-GGGGTTTCTACTGGTGGT-3′) and *amoA*-2R (5′-CCTCGGGAAAGCCTTCTTC-3′) primers^58^. The thermocycling conditions were as follows: 30 s at 95 °C, followed by 40 cycles of 30 s at 95 °C and 34 s at 60 °C, 60 s at72 °C and Crenarchaeal *amoA* CrenamoA23fd: (5′-ATGGTCTGGCTWAGACG-3′) and CrenamoA616rd: (5′-GCCATC CATCTGTATGTCCA-3′)^59^ using 95 °C for 5 min; followed by 10 cycles of 94 °C for 30 s, 55 °C for 30 s, 72 °C for 1 min; followed by 25 cycles of 92 °C for 30 s, 55 °C for 30 s, 72 °C or 1 min; followed by 72 °C at 10 min. The calibration curve was obtained by using a 10-fold serial dilution of a known concentration of positive control DNA (ranging from 10^2^ to 10^8^). The C_T_ values obtained from each sample were then compared with the standard curve to determine the original sample DNA concentration.

### Data analysis

All data were submitted to analysis of variance (ANOVA) for normally distributed variables and Kruskal-Wallis test for non-normally distributed variables (at the 5% probability level). Multiple comparisons among treatments of normally distributed variables were performed using the Tukey test, while for not normally distributed variables, we used the Nemenyi test. All analyses were conducted using the statistics software “R” (v.3.2.2).
